# Whole genome amplification with SurePlex results in better copy number alteration detection using sequencing data compared to the MALBAC method

**DOI:** 10.1038/srep11711

**Published:** 2015-06-30

**Authors:** Lieselot Deleye, Dieter De Coninck, Christodoulos Christodoulou, Tom Sante, Annelies Dheedene, Björn Heindryckx, Etienne Van den Abbeel, Petra De Sutter, Björn Menten, Dieter Deforce, Filip Van Nieuwerburgh

**Affiliations:** 1 Laboratory of Pharmaceutical Biotechnology, Ghent University, Ottergemsesteenweg 460, 9000 Ghent, Belgium; 2 Department for Reproductive Medicine, Ghent University Hospital, De Pintelaan 185, 9000 Ghent, Belgium; 3 Center for Medical Genetics, Ghent University, De Pintelaan 185, 9000 Ghent, Belgium

## Abstract

Current whole genome amplification (WGA) methods lead to amplification bias resulting in over- and under-represented regions in the genome. Nevertheless, certain WGA methods, such as SurePlex and subsequent arrayCGH analysis, make it possible to detect copy number alterations (CNAs) at a 10 Mb resolution. A more uniform WGA combined with massive parallel sequencing (MPS), however, could allow detection at higher resolution and lower cost. Recently, MALBAC, a new WGA method, claims unparalleled performance. Here, we compared the well-established SurePlex and MALBAC WGA for their ability to detect CNAs in MPS generated data and, in addition, compared PCR-free MPS library preparation with the standard enrichment PCR library preparation. Results showed that SurePlex amplification led to more uniformity across the genome, allowing for a better CNA detection with less false positives compared to MALBAC amplified samples. An even more uniform coverage was observed in samples following a PCR-free library preparation. In general, the combination of SurePlex and MPS led to the same chromosomal profile compared to a reference arrayCGH from unamplified genomic DNA, underlining the large potential of MPS techniques in CNA detection from a limited number of DNA material.

Today, massive parallel sequencing (MPS) techniques undergo a rapid and continuous evolution and improvement in accuracy, speed, and cost efficiency. An important factor determining the success of the sequencing of limited amounts of starting material, is the whole genome amplification (WGA) protocol. Bias introduced during this amplification process, may lead to misinterpretations of the genomic profile. Especially when very low amounts of DNA have to be amplified, such as DNA from single cells, some WGA methods will lead to a disproportionate amplification of genomic regions. This results in false positive or false negative copy number changes and allelic dropouts and will be of great importance for applications with the purpose of detecting copy number changes in the genome. An example of such application is pre-implantation genetic diagnosis (PGD) to select an embryo fit for implantation based on the DNA analysis of 4–7 trophectoderm cells. State-of-the-art PGD, using array Comparative Genomic Hybridization (arrayCGH), allows to determine the aneuploidy in the embryo as well as copy number alterations (CNAs), such as deletions, duplications and unbalanced translocations of size larger than 10 Mb. Nowadays, MPS techniques are being introduced in this field^1^,^2^,^3^,^4^ which rises the opportunity to increase the resolution at a reasonable price. Oncogenetics is another field where a faithful analysis of a limited amount of DNA is of great interest. Analyzing the genome of individual cells is important to dissect cancer evolution and to provide the potential to considerably change both cancer research and clinical practice^5^.

A number of commercially available WGA kits have already been individually tested for single cell sequencing, including degenerate oligonucleotide primed PCR^6^ and primer extension PCR^7^,^8^. However, these resulted in allelic drop out (ADO) or preferential amplification of one of both alleles^9^. Another technique, Picoplex/Sureplex (Rubicon Genomics Inc., MI 48108, USA / BlueGnome Ltd., Mill Court, Great Shelford, Cambridge, UK) which is the current standard WGA method for PGD arrayCGH, is based on the use of specific self-inert degenerative primers in the formation of an *in vitro* molecular library that can be amplified by PCR utilizing flanking universal priming sites. Based on the company brochures, an ADO rate limited to 10% can be expected, which is a major improvement over previous PCR-based methods. Recently, a new method, Multiple Annealing and Looping Based Amplification Cycles (MALBAC) (Yikon genomics, Beijing, China) was developed. According to their patent, this method would lead to less amplification bias compared to the SurePlex procedure (WO 2012166425 A2). As the name suggests, loops are formed from the first generated amplicons, which ensures that these amplicons are no longer available as template during this first amplification round. During a second amplification step, these loops will form a more homogeneous template for PCR amplification. In this way, a semi-linear amplification takes place. Ning *et al.* (2014) compared MALBAC with two other WGA methods, Multiple Displacement Amplification (MDA) and a GenomePlex PCR-based method, and concluded that MALBAC had the best genome coverage with excellent reproducibility^10^. In general, it has been shown that each WGA method has its own advantages and disadvantages and that the best method should be selected based on its intended application. A recent article, for instance, suggested that MDA would be better for single nucleotide polymorphism detection (SNP) while MALBAC would be better for CNA detection^11^.

On top of the representation bias introduced by WGA, MPS library preparation can also introduce additional bias due to the enrichment PCR amplification of adapter-ligated fragments. Extra cycles of amplification could lead to extra representation bias in the results.

The goal of this study was to compare commercial SurePlex and MALBAC WGA protocols for their ability to produce optimal MPS data for aneuploidy screening and copy number analysis from a limited number of cells. Samples consisting of 1, 3 or 5 cells, in triplicate, were collected from the LOUCY lymphoblastoid cell line using micromanipulation. The samples were amplified using both WGA methods and Illumina library preparation and sequencing was performed on these WGA products. Subsequent enrichment PCR or PCR-free MPS library preparation was performed in parallel to compare the representation bias. For the different WGA and sequencing library preparation methods, the variability in distribution of the reads across the genome and the ability to correctly detect chromosomal aneuploidies and large CNAs was compared using the bioinformatics tool ReadDepth^12^. The final MPS results were compared to a 180 K arrayCGH profile (Agilent technologies) of genomic DNA of the LOUCY cell line which was determined prior to the experiments to serve as a reference performance standard.

## Material and Methods

### Experimental design

**Figure 1:**
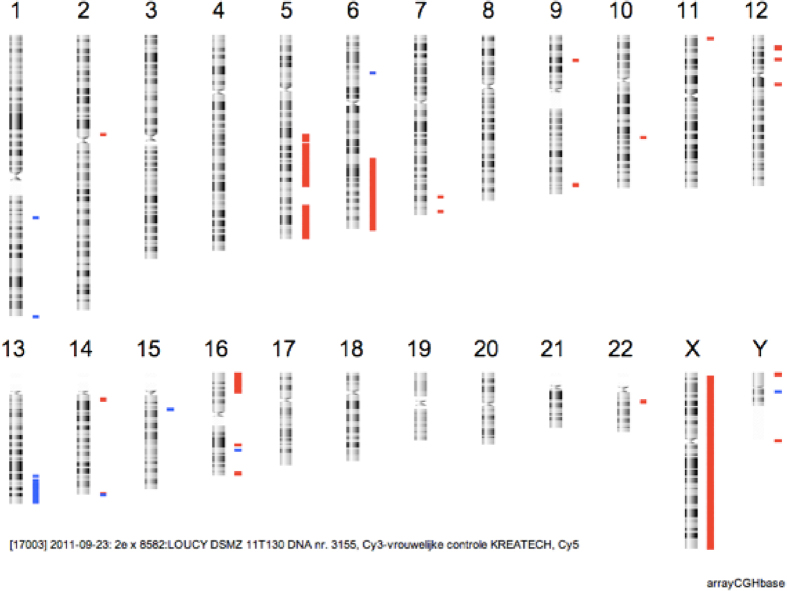
180 K arrayCGH of genomic DNA from the female LOUCY cell line. This profile shows all CNAs detected in the female LOUCY cell line up to a resolution of 50 kb. Red bars indicate deletions and blue bars indicate insertions. The deletions in chromosomes X, 5, 6, and 16 and duplication in chromosome 13, all with a size of >3 Mb, were the ones expected to be detected by the sequencing results. This only accounts for the 1Mb BAC arrayCGH.

**Figure 2:**
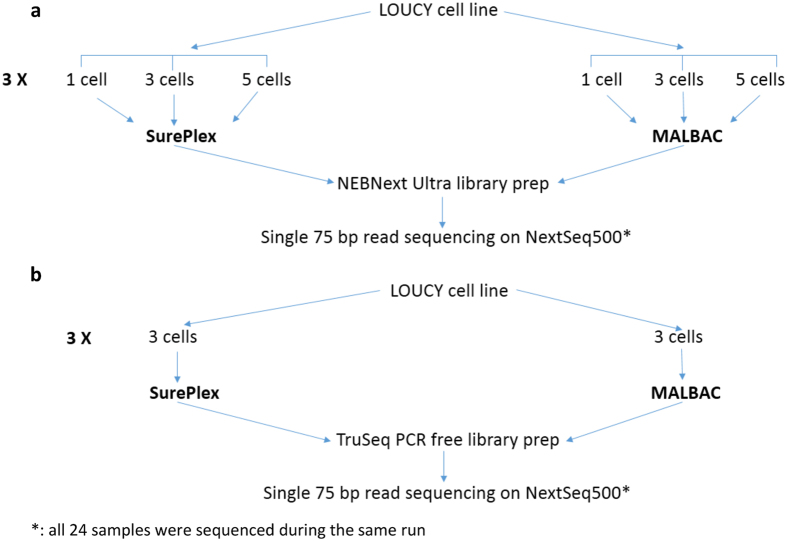
Experimental design : (**a**) Experiment comparing SurePlex and MALBAC WGA methods. (**b**) Experiment to investigate effect of enrichment PCR during library preparation for both SurePlex and MALBAC amplified samples.

This study was performed on cells derived from the female LOUCY cell line (ATCC CRL-2629^13^). A reference 180 K arrayCGH profile (Agilent Technologies) from unamplified genomic DNA from this cell line was obtained prior to the start of the experiments, i.e. before handling and isolation of the samples (Fig. 1). The sequencing results will be compared to this reference arrayCGH profile for CNAs of 3 Mb and larger. The cell line was grown in suspension which allowed isolation of individual cells. The influence of two commercially available whole genome amplification kits (MALBAC and SurePlex) on the ability to detect CNAs in a MPS approach was assessed. For each amplification method, a total of 12 samples was used. Triplicates of samples with 1, 3 or 5 cells were prepared with a standard enrichment PCR based Illumina sequencing library preparation (Fig. 2a). Secondary, triplicates of samples with 3 cells were prepared with a PCR-free Illumina sequencing library preparation (Fig. 2b).

### Growth and isolation of cells

The cells were grown in Roswell Park Memorial Institute (RPMI-1640) medium (Life technologies, Carlsbad, USA), supplemented with 10% fetal bovine serum (Life technologies, Carlsbad, USA). For optimal growth they were kept at a temperature of 37 °C, a 5% CO_2_ and 95% O_2_ level. A known amount of cells was isolated with an ergonomic denuding handle from STRIPPER® (Origio, Måløv, Denmark) and MXL3-100 needles with a diameter of 100 μm (Origio, Måløv, Denmark). A serial dilution with sterile phosphate buffered saline (PBS) (Life technologies, Carlsbad, USA) spots to the desired amount of cells for isolation was performed on a Petri dish (5.5 cm) under an Axiovert 25 light microscope (Zeiss, Jena, Germany). All cells were collected in a maximum volume of 1 μl for the MALBAC samples and 2.5 μl for the SurePlex samples, as this is required for optimal lysis of the cells, according to manufacturer’s instructions. Immediately after collection, all samples were snap frozen in liquid N_2_.

### MALBAC WGA

Cell lysis and amplification was performed, using the *MALBAC kit* (Yikon genomics YK001A/B version 1302.1, Jiangsu, China), following manufacturer’s instructions. As a positive control, 1 μl of male control DNA (9948; Promega; 10 ng/μl) was used at a concentration of 30 pg/μl. The blank was 1 μl of PBS. All samples were purified according to the manufacturer’s protocol of the *Genomic DNA Clean & Concentrator*^*TM*^ *kit* (version 1.0.0, Zymo Research, Irvine, USA) with 5 X binding buffer. Concentration was measured using *Qubit® dsDNA High Sensitivity Assay kit* (Life technologies, Carlsbad, USA).

### SurePlex WGA

Cell lysis and amplification was performed following manufacturer’s instructions using the *SurePlex Amplification system* (Bluegnome, Cambridge, United Kingdom). As a positive control, 2.5 μl of female control DNA (G1521; Promega; 187 ng/μl) was used at a concentration of 25 pg/μl. The blank was equal to 2.5 μl of PBS. Purification and concentration measurements were done in the same way as described above.

### ArrayCGH

One of the three cell samples, amplified by SurePlex, was also analyzed using a 1 Mb BAC array (Bluegnome) (Supplementary fig. S1 online). The CNAs observed on this array should also be observed on the reference array profile. The arrayCGH was performed according to the *24Sure* Protocol (Bluegnome) with a male genomic DNA sample as reference.

### Illumina library preparation

Hundred ng of the WGA products was fragmented to an average size distribution of 200 bp with the *S2 Focused Ultrasonicator with Adaptive Focused Acoustics (AFA) technology* (Covaris, Woburn, USA). All samples were diluted in Tris-EDTA buffer (TE-buffer) to a volume of 130 μl in microTUBES (Covaris, Woburn, USA). The programmed guidelines for fragmentation to 200 bp were followed (Duty cycle of 10%, Intensity of 5 and 200 cycles/burst), but the fragmentation time was prolonged to 190 sec based on previous experience.

Subsequently, libraries of the fragmented samples were created using *NEBNext® Ultra™ DNA Library Prep* (Chapter 2B, New England Biolabs, Ipswich, USA), following manufacturer’s protocol with modifications as described here. After incubation with the USER enzyme, a DNA purification step (*Zymo Genomic DNA Clean & Concentrator*^*TM*^) was included before the size selection step. Size selection was performed with the E-Gel iBase Power system (Invitrogen) using an E-gel EX 2% agarose gel and a 1 kb Plus DNA ladder (Thermo Fisher Scientific, Waltham, USA). For all samples, fragments with a size of ~300 bp were cut from the gel and DNA was recovered using the *Zymoclean gel DNA recovery kit* (Zymo research). The size selected DNA samples were then subjected to an enrichment PCR using *NEBNext® Multiplex Oligos for Illumina® (Index Primers Set 1 and 2)* according to the protocol, with addition of tRNA to minimize the loss of DNA via tube interaction. The quality of the different samples was assessed with the *Agilent High-Sensitivity DNA kit* (Bioanalyser, Agilent Technologies, California, USA).

The PCR-free libraries were created entirely according to the *TruSeq DNA PCR-free LT sample preparation kit* (Illumina), which does not require an enrichment PCR. For each sample, the entire amount of WGA product was used as starting material, which was on average 700 ng. This is lower than the 1 μg input required by the protocol. From here onwards, these samples are called ‘PCR-free’ samples.

Before sequencing the samples, the amount of sequence-able library fragments was determined by performing a qPCR according to the *Sequencing Library qPCR Quantification* kit (Illumina, San Diego, USA). Samples were diluted to 10 nM with elution buffer (EB buffer) (QIAGEN, Hilden, Germany). The control template used for the standard curve was a PhiX control library (10 nM).

Finally, single-end index 75 bp sequencing was performed on a high output flowcell on a NextSeq500 (Illumina, California, USA). 24 samples with each a different index were multiplexed on one flowcell. Samples were pooled at 2 nM each and diluted to a final concentration of 12.1 pM. Sequencing of 24 samples at 75 bp on a NextSeq500 should lead to an average genome coverage of 0.4X per sample (16M reads/sample).

### Data analysis

The FastQ files imported from Basespace (Illumina), were quality controlled using FastQCv0.10.1 (Babraham bioinformatics). This revealed a disproportionate oscillation of the percentage of the bases in the first 30 bp of the reads. The disproportionate oscillation in the percentage of bases was more pronounced in the SurePlex amplified samples (Supplementary fig. S2 online). In addition to trimming these 30 bp, TruSeq adapters and low quality read ends with Phred quality score <20 were trimmed with Cutadapt v1.4.2.

Reads with minimum length of 30 bp were subsequently aligned to the human genome hg19 using the Burrow-Wheeler-Aligner (BWA) v0.7.5a algorithm and non-uniquely mapping reads were removed with SAMtools v0.1.19 and BEDtools v2.17.0^14^,^15^. CNA detection was performed using the bioinformatics tool ReadDepth^12^, which performed best in a recent comparison of different CNA detection methods^16^. For this, the genome sequence was divided into non-overlapping windows or bins of 1 Mb in size and the number of reads per bin was calculated. The number of reads per bin was corrected for bias introduced by the inability to map reads into repetitive regions of the genome by using mappability tracks created via self-alignment of the reference genome. Additionally, the mappability corrected read numbers per bin were normalized for GC-content. For this, the average read depth for bins with GC content was calculated in intervals of 0.1% before using LOESS smoothing to fit a regression line to this data. The number of reads per bin was scaled using a correction value equal to the difference between the median read depth and the average read depth of that bin and in such way that the correction was neutral with respect to the total number of reads. Finally, bins were median normalized. Bins with a similar amount of reads were detected using a circular binary segmentation algorithm (alpha value of 0.01 and min.width value of 2) and considered as one segment. Based on an estimated amount of copy-number gain or loss in the genome (both 5%; standard settings determined by the authors of the ReadDepth tool yielding a good performance^12^) and an overdispersion factor equal to 5 (for which the observed and modeled distributions were most similar), three different negative binomial distributions were modeled for monosomic, diploid or trisomic regions, respectively. The two values at which the three peaks of these distributions were maximally separated were considered as thresholds for monosomic and trisomic regions. Estimated copy numbers for segments that exceeded these thresholds were flagged as CNAs. Copy numbers were estimated under the assumption that for a diploid genome the majority of the genome is copy number two. The copy number of other regions was subsequently estimated based on the segment ratio relative to two.

Comparing the yield after amplification and library preparation between the different groups was performed using t-test statistics. The variance in number of reads per 1 Mb bin across the genome was compared between MALBAC, SurePlex and the PCR-free samples by means of a mean-scaled first order estimator. In more detail, GC-content and mapability normalized count data per bin was divided by the average number of reads per bin, thus correcting for possible differences in sequencing depth. We then estimated the variance across bins as the running sum of the squared difference between the mean scaled number of reads of one bin and the previous bin divided by the total number of bins, which is the same for all samples. The variance due to counting statistics in profiles normalized so that the mean value is 1.0, is equal to 1/N, where N is the average number of reads per bin (neglecting small effects due to copy number aberrations and counting corrections). As such, the difference between the variance of the copy number profile, estimated by the first-order estimator, and the variance due to counting statistics (1/N) for that profile gives a measure of the noise contribution from the entire sample handling, preparation and analytical process, independent of sequencing depth. The difference in percentage of reads mapped in the different groups was analyzed by a one-way ANOVA on the arcsine transformed data, followed by a Bonferroni’s multiple comparison post-hoc test. When the prerequisites for valid ANOVA testing could not be met, an ANOVA on ranks followed by post-hoc Dunn’s method was performed instead. P-values  <0.05 were considered significant. Numbers given after ‘±’ symbol in results indicate standard deviation. The sensitivity was defined as the number of true positive calls divided by all positive results for the cell line (true positive + false negatives). The positive predictive value (PPV) was defined as the number of true positive calls divided by the number of positive calls (true positive + false positive). Raw sequencing data are deposited in the NCBI Sequence Read Archive under project accession number SRP051311.

## Results

### Evaluation of LOUCY cell line stability

As a reference for our DNA samples, a 180 K arrayCGH profile of genomic DNA of the LOUCY cell line was used (Fig. 1). The 180 K arrays have an average resolution of ~50 kb. The resolution aimed by the MPS analysis is to find CNAs of 3 Mb in size or larger. This resolution was chosen in view of performing PGD using MPS. Currently the resolution for PGD is 10 Mb. However, some small CNAs are not well-defined at this resolution. Opting for a higher resolution could include these CNAs as well. 3 Mb was considered a minimum because the variation caused by the WGA was a limiting factor at lower bin sizes. BAC arrayCGH analysis was performed on amplified DNA at a resolution of 10 Mb (Supplementary fig. S1 online). If the 180 arrayCGH profile was filtered for CNAs > 3 Mb in size, both profiles showed a similar pattern, confirming that the cell line was not altered after manipulation. According to the 180 K arrayCGH profile, the following chromosomal aneuploidies and CNAs were called within the resolution range of the subsequent sequencing results (>3 Mb): a deletion of an entire X-chromosome, a distal deletion of ±72 Mb on 5q21.3q35.3, a distal deletion of ±45 Mb on 6q22.31q27, a ±3 Mb duplication of 13q33-q33.3 and deletions of respectively 13 Mb and 3 Mb on 16p13.3-p13.12 and 16q24.2q24.3. These CNAs were used as a reference for comparison with the MPS analyses.

### Yield after WGA and library preparation

**Figure 3:**
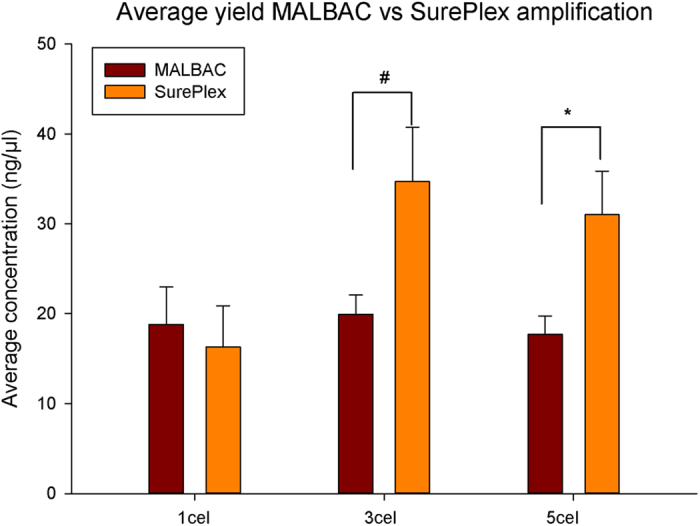
Average yield after MALBAC and SurePlex amplification for the different amounts of starting material used. The difference between MALBAC and SurePlex within each group was tested with a t-test. One-tailed p-values are #p = 0,000305; *p = 0,00352. 1 cell N = 6; 3 cell N = 9; 5 cell N = 6.

Figure 3 compares the WGA product yield of both tested WGA methods. The final volume of all samples was the same. Both WGA methods resulted in a similar yield after amplification of the samples containing 1 cell. The yield after amplification with SurePlex was significantly higher than the yield after MALBAC amplification when started from samples of 3 (34.7 ± 6.1 ng/μl vs. 19.9 ± 2.1 ng/μl) or 5 cells (31 ± 4.8 ng/μl vs. 17.7 ± 5 ng/μl).

For the libraries that were prepared using enrichment PCR, there was no significant difference in the amount of sequenceable fragments measured by qPCR for MALBAC or SurePlex pre-amplified samples (14.8 ± 3.7 nM and 18.5 ± 6.3 nM for MALBAC and SurePlex amplified samples, respectively). In contrast, for the PCR-free library preparations, a significant difference was detected between the samples with MALBAC (1.1 ± 0.6 nM) and SurePlex pre-amplification (4.6 ± 1.5 nM).

### Sequencing quality

With a density of 140 K/mm^3^ and a total (passed-filter) read count of 324.76 M, the run quality was within expectations with 87% passing the quality threshold of 30, a Full width at half maximum (FWHM) of 3 and a 1.66% alignment of the PhiX control. After adaptor and quality trimming and mapping of the reads, an average of 12,708,048 reads of ±30 bp was mapped per sample, resulting in an average coverage of 0.1X per sample. The PCR-free SurePlex prepped samples had a lower number of reads mapped, compared to the other samples. While all other samples contributed on average to 14,013,601 reads per sample, these samples only had 3,569,175 reads on average mapped per sample (coverage of 0.03X). To allow comparison between PCR-free libraries and enrichment PCR amplified libraries, the latter were randomly down-sampled to a comparable raw read count.

### Difference in read mapping between MALBAC and SurePlex

**Figure 4:**
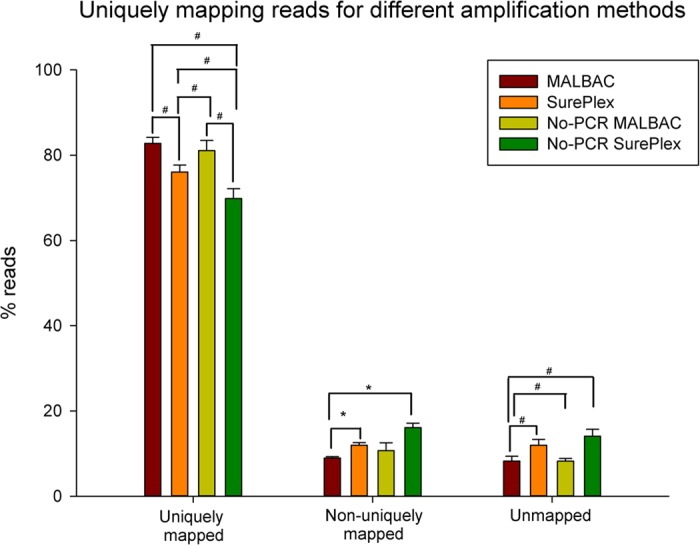
Scheme of read mapping after different amplification methods. MALBAC amplified samples have more uniquely mapped reads compared to SurePlex amplified samples. Samples were combined for the different starting amounts. Statistical analysis was performed with a one-way ANOVA followed by a Bonferroni t-test for multiple comparison. When comparing the percentages of non-uniquely mapped samples, the equal variance test failed. Here an ANOVA on ranks was performed followed by Dunn’s method for multiple comparison. #p < 0.001 and *p < 0.05.

When comparing the mapping results within each protocol from the samples with a different number of input cells, no significant differences were detected in the relative number of uniquely, non-uniquely and unmapped reads (p>0.05). A significantly higher percentage of reads, generated from the samples amplified with MALBAC, mapped uniquely compared to the percentage of uniquely mapped reads from samples amplified with SurePlex (82.8 ± 1.4% vs 76.1 ± 1.6%, respectively) (Fig. 4). This also holds true for the MALBAC and SurePlex amplified samples that were not enriched by PCR during library preparation (81.1 ± 2.4% vs 69.8 ± 2.3%, respectively). The percentage of uniquely mapped reads was significantly higher in the SurePlex amplified samples compared to the SurePlex amplified samples prepared without enrichment PCR. No significant difference was found for the MALBAC samples prepared with enrichment PCR versus the PCR-free MALBAC amplified samples.

The distributions of the GC content per read showed for both MALBAC and SurePlex amplified samples a mean GC-content higher than the GC-content in the human genome, around 46% and 45% vs 42.4% for MALBAC and SurePlex, respectively (Supplementary fig S3 online). Omission of the enrichment PCR during library preparation resulted for both amplification protocols in slightly lower average GC-contents (43% for both MALBAC and SurePlex), yet the distributions were less well-shaped (Supplementary fig S3 online).

### Read distribution across the genome

The variance in read distribution across the genome was significantly different between MALBAC and SurePlex amplified samples. The number of reads per 1 Mb bin in MALBAC amplified samples varied more across the genome as compared to SurePlex amplified samples. This was the case for 1 cell, 3 cells and 5 cell samples (Table 1). The variability in mapping had the consequence that a lot of bins, in the MALBAC amplified samples, fell outside of the range expected for diploidy, even in regions where the LOUCY genome is diploid. This was not observed in the SurePlex amplified samples, were a more smooth read distribution was observed.

### Detection of chromosomal aneuploidy and copy number variants

**Figure 5:**
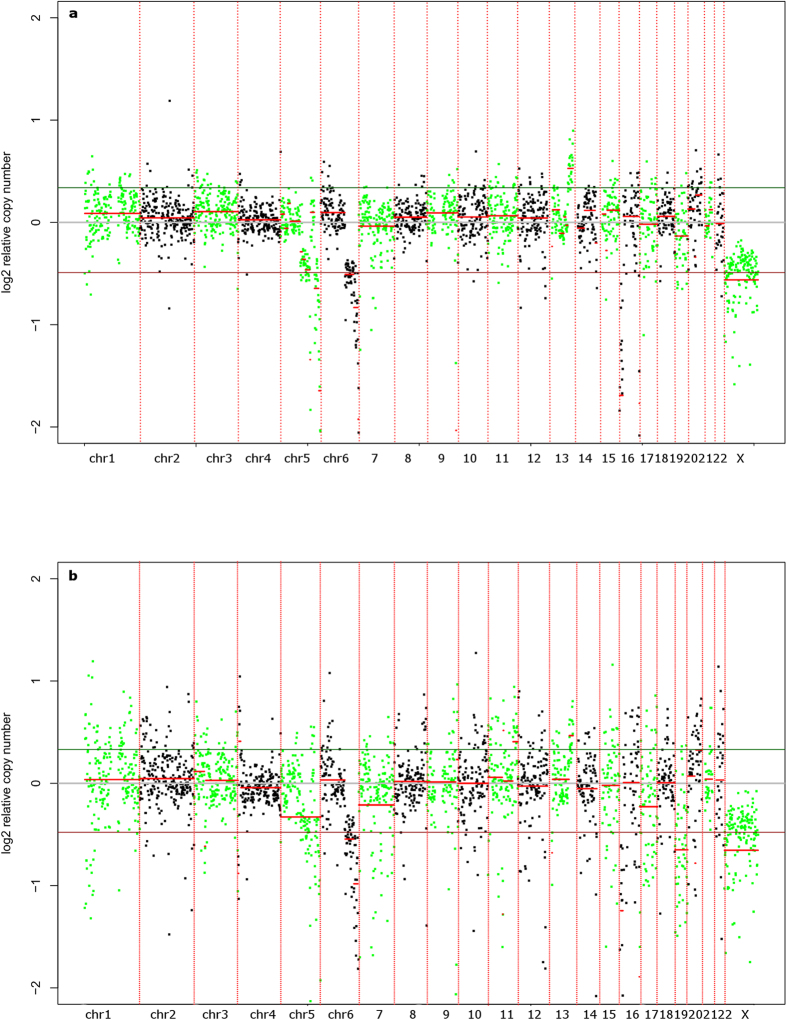
Samples amplified with SurePlex are better suited for detection of aneuploidies and CNAs. **a**) All arrayCGH-verified deletions in chromosomes X, 5, 6, 9 and 16 and duplication of 13 are only detected in SurePlex amplified samples. (**b**) MALBAC amplified samples show, because of their bigger variability, false-positives. Each dot on the plots represents an equally sized bin of 1 Mb. Separate chromosomes (from 1 to 22 and X) are colored green/black alternatingly. The X-axis indicates log_2_(copy number/2), estimated based on the number of reads per bin. A log_2_(copy number/2) equal to zero corresponds to a copy number of 2, as would be expected for a diploid genome. Theoretically for an insertion a log_2_ (copy number/2) of 0.5 is expected, whereas for a deletion a log_2_ (copy number/2) of -1 is expected. The red and green horizontal lines represent the threshold for detection of insertion or deletion, respectively. The brighter red line combines the bins with equal means.

The first way to detect CNAs is using an algorithm such as used by ReadDepth. Next to this, CNAs can also be represented as a graphical profile (Fig. 5), allowing a more manual interpretation. The choice for 1 Mb bins allowed us to detect CNAs at least 3 Mb in size. Analysis of the MALBAC amplified samples resulted in many false positive CNA calls random across the genome, which were not observed on the reference arrayCGH profile (Table 2; Supplementary Table 1 online). Only one of the MALBAC amplified samples resulted in detection of all CNAs that were present according to the arrayCGH reference (Table 2; Supplementary Table 1 online). Most of the samples missed more than one of the expected CNAs. On the other hand, of all samples amplified with SurePlex, only two gave an incomplete detection of the CNAs and only a few false positive CNAs were detected. Figure 5 shows the CNA profiles of two 3-cell samples amplified with either SurePlex (Fig. 5a) or MALBAC (Fig. 5b). The profile of the SurePlex samples is smoother and the expected CNAs are clearly observed. The CNA profiles of all samples used during this experiment are shown in Supplementary fig. S4 online. These results show a sensitivity for calling the correct CNA equal to 59.3% (±22.2%; mean ± standard deviation) for MALBAC amplified samples and 94.4% (±11.8%) for SurePlex amplified samples. The difference between both methods, is also reflected in the PPV, with a PPV of 42% (±12.3%) and 77.7% (±11.8%) for MALBAC and SurePlex amplified samples, respectively.

### The influence of a PCR-free library preparation on read distribution and CNA detection

**Figure 6:**
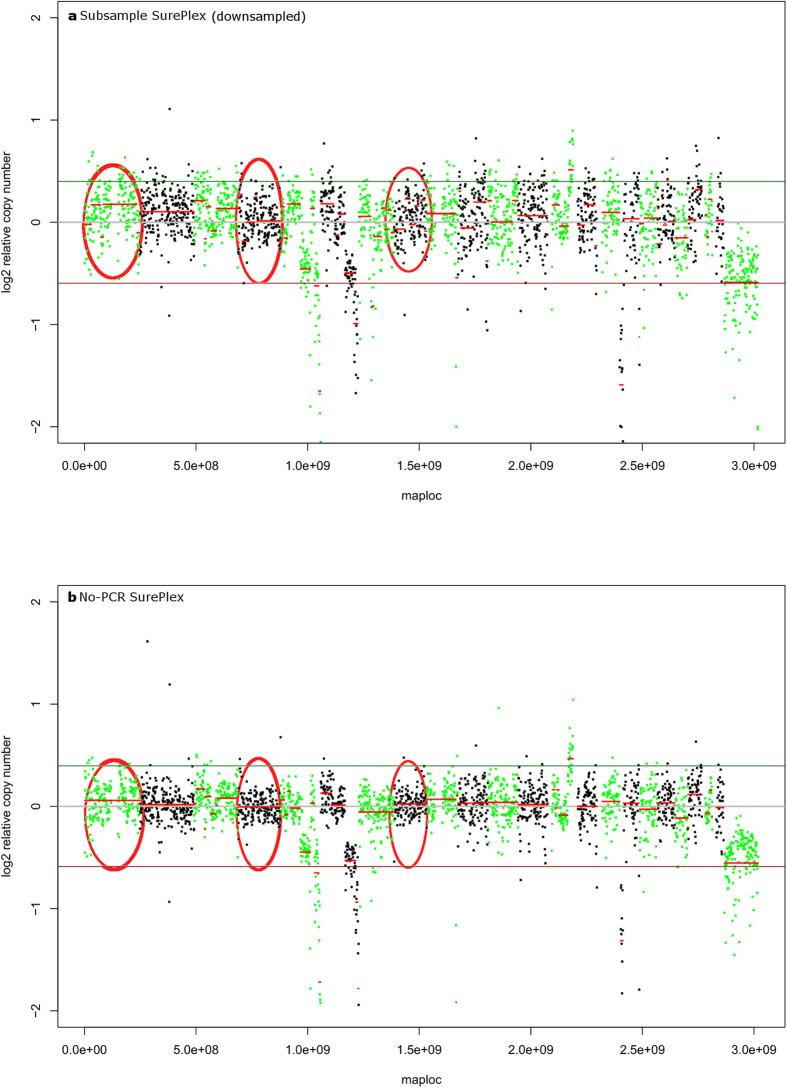
Omitting enrichment PCR during library preparation leads to smoother CNA profiles. As a result of the decreased variation, copy number estimation is more consistent across chromosomes, for instance in chromosome 1, 4 and 8 (red circles). This will be an important benefit if screening at higher resolutions is performed. To allow direct comparison, ‘SurePlex PCR’ samples were randomly downsampled to obtain a similar number of raw reads as ‘SurePlex no-PCR’ samples.

The variance in read distribution across the genome was significantly different between enrichment PCR amplified and PCR-free samples. The number of reads per 1 Mb bin in the PCR-free SurePlex samples varied less across the genome compared to enrichment PCR amplified SurePlex samples (Table 1). The detection of CNAs was comparable at this resolution, yet slightly more consistent in PCR-free SurePlex samples compared to enrichment PCR amplified SurePlex samples (Fig. 6). Although the X-chromosome monosomy was not called by ReadDepth, on the graphical profiles it can be distinguished. MALBAC amplified samples with subsequent PCR-free library preparation also resulted in improved MALBAC variation, but still a lot of false positive CNAs were detected in these libraries. CNA calling in SurePlex-amplified samples with PCR-free library preparation has a PPV of 100% (±0%; mean ± standard deviation) and a sensitivity of 77.8% (±9.6%). CNA calling in the MALBAC amplified PCR-free library preparation samples has a PPV of 50% ± 26.8% and a sensitivity of 50% ± 33%.

## Discussion

SurePlex amplification has already proven its efficiency in combination with the 24Sure arrayCGH protocol for PGD purposes. During arrayCGH, amplification bias could partially be overcome by using the same whole genome amplification (WGA) method for both sample and reference. However, due to the rather random nature of the bias introduced by WGA, the possibility of bias cannot be completely eliminated^17^. Because of the nature of the detection of aneuploidies and CNAs from MPS data, it is not necessary to sequence a reference with the samples every time. This, however makes it even more important to obtain an unbiased amplification. De Bourcy *et al.* (2014), observed that PCR-based methods like MALBAC and NEB-WGA, are better suited for CNA detection than MDA methods because of their low variability. NEB-WGA is similar to the SurePlex WGA method used in this study, while the MALBAC WGA method of Bourcy *et al.* (2014) is comparable, yet not entirely similar, to the MALBAC WGA method used in this study. In the present study, MALBAC (Yikon Genomics) and SurePlex (Bluegnome), both commercial kits, were compared for their usability in CNA detection in a more complex, say diploid human genome, setting that has to our knowledge not been done before.

The reference arrayCGH used for this study was performed with unamplified genomic material, thus avoiding the bias introduced by WGA. The resolution aimed by the sequencing during this study, is in the range of state-of-the-art PGD with arrayCGH. With 1 Mb bins, CNAs of 3 Mb and larger should be successfully detected by ReadDepth. However, as stated in the results, some smaller CNA can also be called if the variation in that specific region is low. We were, for instance, able to detect a deletion of 2.5 Mb at chromosome 9 (q34.11q34.12), which was also detected, and thus confirmed, on the 180 K and 1 Mb arrayCGH. In the sequencing results this particular deletion was called as a 3 Mb CNA because sequencing is only accurate down to 1 Mb.

Based on raw read count, an average genome coverage of approximately 0.3x was obtained for most samples. This theoretical calculation of the coverage gives an indication of how much of the human genome could theoretically be covered with a certain number of reads of a given length. However, after adaptor and quality trimming and mapping of the reads, an actual coverage of approximately 0.1X was obtained, except for the PCR-free SurePlex samples, for which a coverage after mapping of only 0.03X was obtained. It was shown that MALBAC amplified DNA samples yielded a significantly higher amount of uniquely mapped reads compared to SurePlex amplified samples. If these uniquely mapped reads are distributed uniformly along the genome, this would indicate that the MALBAC amplified samples cover the reference genome better, potentially increasing the accuracy in which CNAs can be detected. However, the distribution of the reads along the different bins in every chromosome showed not to be uniform for the MALBAC amplified samples (Table 1). Indeed, the variance in read counts per 1 Mb bin across the genome was significantly higher compared to the SurePlex samples, suggesting that certain loci in the genome must be over- or under-amplified during MALBAC amplification.

MALBAC has been reported to have strong GC biases^10^. Indeed, when studying the distribution of the GC-content per read, MALBAC seems to prefer more GC-content rich regions for amplification. Yet, this bias seems also to be prevalent in the reads originating from SurePlex amplified samples. Enrichment PCR during library preparation seems to amplify this GC bias effect, i.e. GC-bias is less pronounced in samples that did not undergo enrichment PCR compared to samples that did. However, where usually a clear Gaussian distribution can be expected, the distribution for the samples that were not enriched by PCR is less well-shaped, i.e. no clear maximum/mean can be distinguished.

Bias caused by over- or underrepresentation of certain parts of the genome, will most likely lead to false interpretations of the copy number status of chromosomal regions. This means that the variability in read distribution across the whole genome is directly related to the suitability to detect CNAs. The smaller the variation, the bigger the possibility that a value lying outside the threshold barriers is a true CNA. The thresholds for diploidy were redefined for every sample based on their negative binomial distribution, but did remain comparable between the samples. Variations in copy number were called if the number of alleles exceeded these boundaries. For only one of the MALBAC amplified samples, the MPS-CNA profile was similar to the reference arrayCGH profile. For SurePlex amplified samples on the other hand, all the expected CNAs were detected in the majority of the samples (7 out of 9). The variability between different bins in the MALBAC samples was too high to distinguish whether regions were actually underrepresented because they were deleted in the original genome or because of amplification bias. In addition, for the MALBAC amplified samples, quite some false positive results were called, that were not visible on the reference array. For instance, chromosome 19 aberrations, a false positive recurrently appearing on arrayCGH^18^, was called for almost all MALBAC amplified samples, but for none of the SurePlex amplified samples. Moreover, calls made for the MALBAC amplified samples were not very consistent across the three replicates, especially for 1 and 3 cell samples. Although such inconsistency was also observed across the replicates of the 1 cell samples amplified with SurePlex, less false positive calls were made compared to the MALBAC amplified samples. In the two cases that not all expected CNAs were called, the correct profile could still be deducted from the graphical profile. The opposite does also occur, where in one SurePlex amplified 3 cell sample a duplication in chromosome 20 was called by ReadDepth, but on the graphical profile this could not be observed. These observations suggest that for good practices both calls made by the ReadDepth algorithms and manual visual inspection of the graphical profiles should be considered. Nowadays, arrayCGH profiles in PGD are interpreted and corrected based on recurrent artifacts, i.e. aberrations that are present in most profiles but which are known not to have any impact on the correct development of the embryo. Such artifacts may result from the used technique and are frequently observed around telomeric and centromeric regions of the chromosome. In this study however, information of such artifacts was not available and thus not taken into account to correct the calls. Nevertheless such information could be obtained by sequencing multiple normal, non-aberrant genomes and screening for recurrent aberrations. In general, this study shows that even without a reference genome the expected calls can be made, although a few false-positives were also called.

The inconsistency observed between different replicates of 1 cell samples could possibly be explained by the cell cycle status of the amplified cells. More evidence is growing that cells that are in S-phase give a more scattered profile because of the replication status of the DNA^19^. Samples containing multiple cells could contain cells that are in a different cell cycle status, which diminishes the influence of the individual cell status.

In the PCR-free MALBAC and PCR-free SurePlex amplified samples, similar overall differences could be observed between both WGA methods. The PCR-free MALBAC amplified samples had again a significantly higher amount of uniquely mapped reads but at the same time also a significantly higher variance in read counts per bin across the genome. This suggests that the enrichment PCR did not cause the difference observed between the two amplification methods. However, omitting the PCR shows an even lower, significant, variance in read distribution across the genome. Still, the difference with the normal diploid parts on the graph were obvious. Also the graphical profile is smoother without the use of enrichment PCR. This might become even more important to detect CNAs at a higher resolution and thus smaller bin size. Generally, these results suggest that at least part of the bias in MPS data for CNA detection could be eliminated by avoiding an enrichment PCR during library preparation.

Although the detection of CNAs was performed with ReadDepth, other bioinformatics tools exist for calling CNAs without the need of a reference genome. Some of them are described in the review of Duan *et al.*, who compare six different tools in different settings^16^. ReadDepth seemed to perform best in most of the conditions tested in this review^16^. However, the tool described by Baslan *et al.*, which should be more specific for single cell analysis^20^, was not considered in this review. Although this tool is quite similar to ReadDepth, it might be expected that in a setting with higher resolution, and thus much less reads per bin, where the bias introduced by WGA will be more prominent, the tool might perform better in calling CNAs compared to ReadDepth. Another recently published tool, qDNAseq, might also have that advantage over ReadDepth, as it uses a blacklist to filter chromosomal regions with anomalous behavior, such as satellites, centromeric and telomeric repeats, reducing the noise level of the data^21^.

## Conclusion

In conclusion, we observed that for CNA detection, the SurePlex protocol is better suited than the MALBAC procedure. MALBAC amplified samples show a high non-uniformity across the genome, which leads to false positive CNAs. This is especially true when starting from single cells. SurePlex amplification has shown its value for single cell chromosomal CNA detection, although for PGD purposes a minimal 3 cell starting amount is recommended. It has also been shown that omitting enrichment PCR during library preparation, will lead to a more uniform coverage of the genome. This may become more important if sequencing depth is being increased to increase resolution.

## Additional Information

**How to cite this article**: Deleye, L. *et al.* Whole genome amplification with SurePlex results in better copy number alteration detection using sequencing data compared to the MALBAC method. *Sci. Rep.* **5**, 11711; doi: 10.1038/srep11711 (2015).

## Supplementary Material

Supplementary Information

## Figures and Tables

**Table 1:** Estimates of variance (mean and standard deviation) and statistical differences in variance for the different tested conditions.

Estimates of variance		
condition	mean	standard deviation
MALBAC 1 cell	0.165	0.037
MALBAC 3 cells	0.138	0.012
MALBAC 5 cells	0.146	0.010
MALBAC no PCR	0.120	0.015
SurePlex 1 cell	0.083	0.013
SurePlex 3 cells	0.077	0.012
SurePlex 5 cells	0.073	0.009
SurePlex no PCR	0.064	0.004
		
Statistical differences in variance		
comparison	p-value	
MALBAC 1 cell vs SurePlex 1 cell	0.0239	
MALBAC 3 cells vs SurePlex 3 cells	0.0031	
MALBAC 5 cells vs SurePlex 5 cells	0.0007	
MALBAC no PCR vs SurePlex no PCR	0.0032	

“no PCR” indicates the absence of an enrichment PCR during library preparation.

**Table 2:** Number of expected calls and false-positives made by all samples.

						

Expected calls were: deletions in chromosomes X, 5, 6, 9 and 16 and duplication in chromosome 13 as expected by array. Calls were of 3 Mb and bigger expected. Calls were summed for the samples that started with the same amount of cells.
