# Supplementary material for: Whole genome amplification with SurePlex results in better copy number alteration detection using sequencing data compared to the MALBAC method
Source: Sci Rep. 2015 Jun 30;5:11711. doi: 10.1038/srep11711 (PMC4485032; doi:10.1038/srep11711)
Supplement: Supplementary Information [file srep11711-s1.pdf]

Whole genome amplification with SurePlex results in better copy number variation alteration using sequencing data compared to the MALBAC method

Lieselot Deleye[1,#,*], Dieter De Coninck[1,#], Christodoulos Christodoulou[2], Tom Sante[3], Annelies Dheedene[3], Björn Heindryckx[2], Van den Abbeel Etienne[2], Petra De Sutter[2], Björn Menten[3], Dieter Deforce[1,$], Filip Van Nieuwerburgh[1,$]

([#,$] contributed equally; * corresponding author)

[1.] Laboratory of Pharmaceutical Biotechnology, Ghent University, Ottergemsesteenweg 460, 9000 Ghent, Belgium
[2.] Department for Reproductive Medicine, Ghent University Hospital, De Pintelaan 185, 9000 Ghent , Belgium
[3.] Center for Medical Genetics, Ghent University, De Pintelaan 185, 9000 Ghent , Belgium

Contact information:
Lieselot Deleye
Ghent University, Laboratory of Pharmaceutical Biotechnology
Ottergemsesteenweg 460, 9000 Ghent, Belgium
lieselot.deleye@telenet.be

Supplementary figure S1

1 Mb arrayCGH profile of the LOUCY cell line determined from a 3 cell sample amplified with SurePlex. The profile confirms the profile determined prior to the start of the experiments. CNAs of 3Mb and more can be detected on this profile.

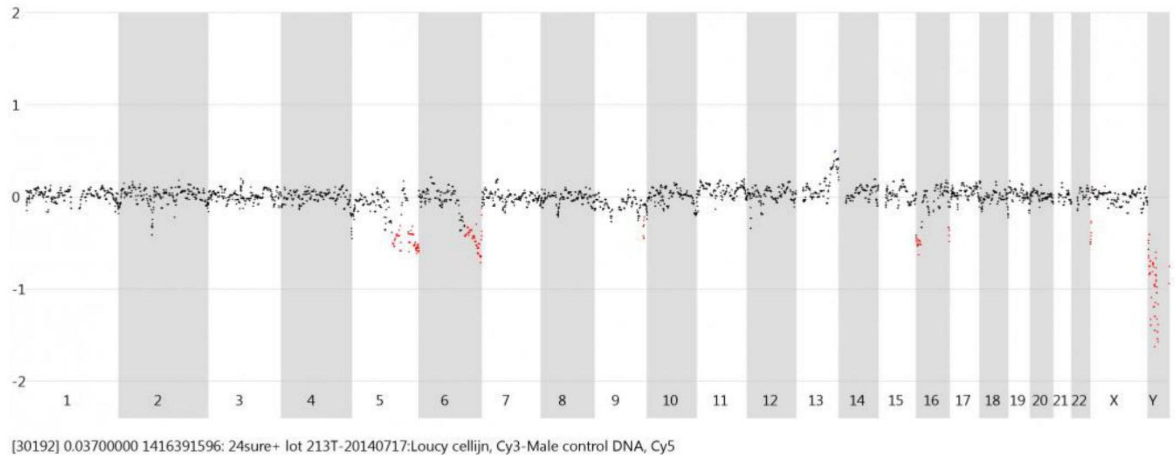

Supplementary figureS2

Sequence content across all bases

The percentage of each base at the different bp positions on the reads is shown in this graph. A) For the MALBAC samples a slightly irregular pattern is observed for the first 30 bases. The last base shows a skewed pattern and will be trimmed. B) The SurePlex samples have a very irregular pattern for the first 30 bases indicating that the adapter probably consists of mainly G and T. This passage, together with the last base, will be trimmed.

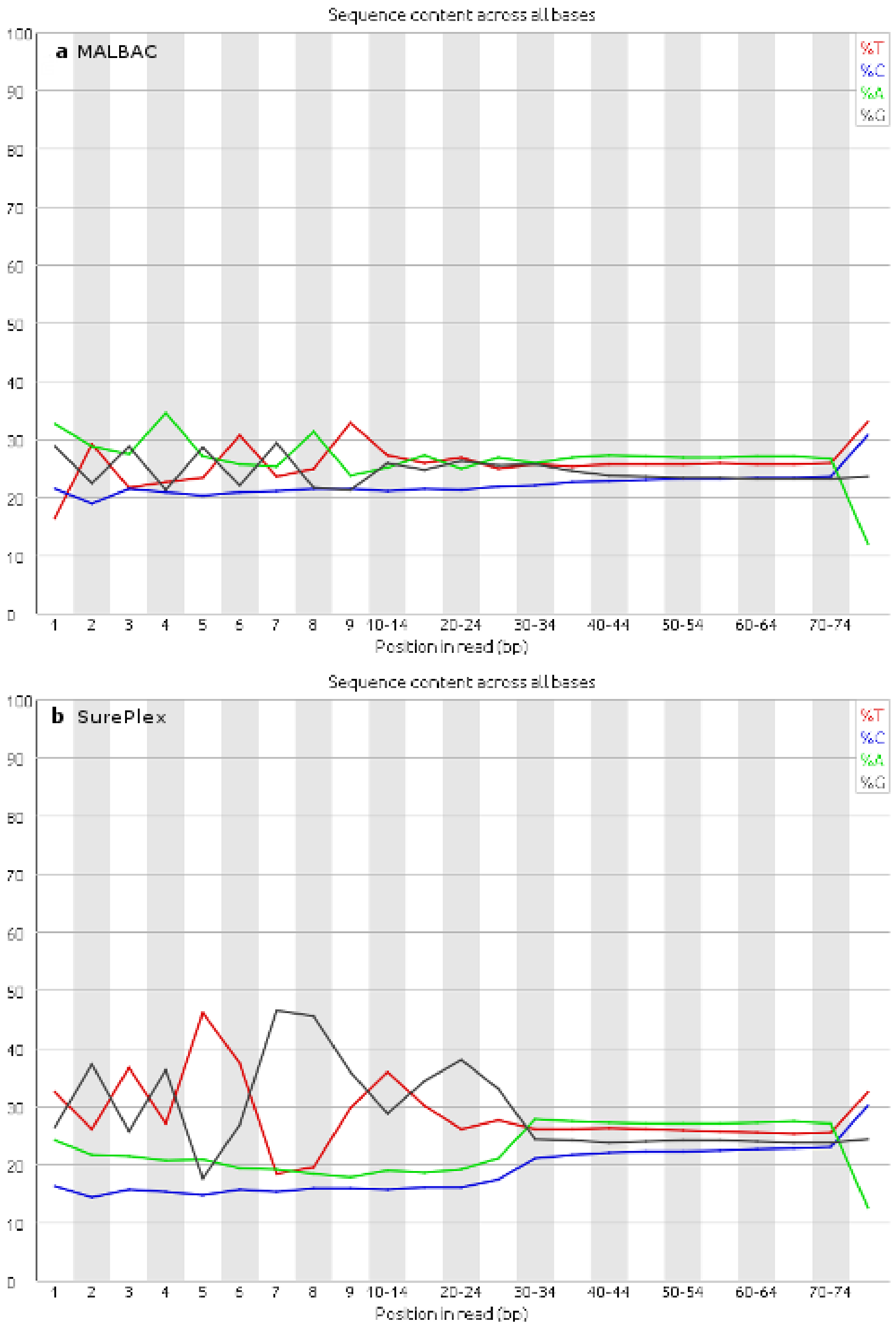

Supplementary figure S3

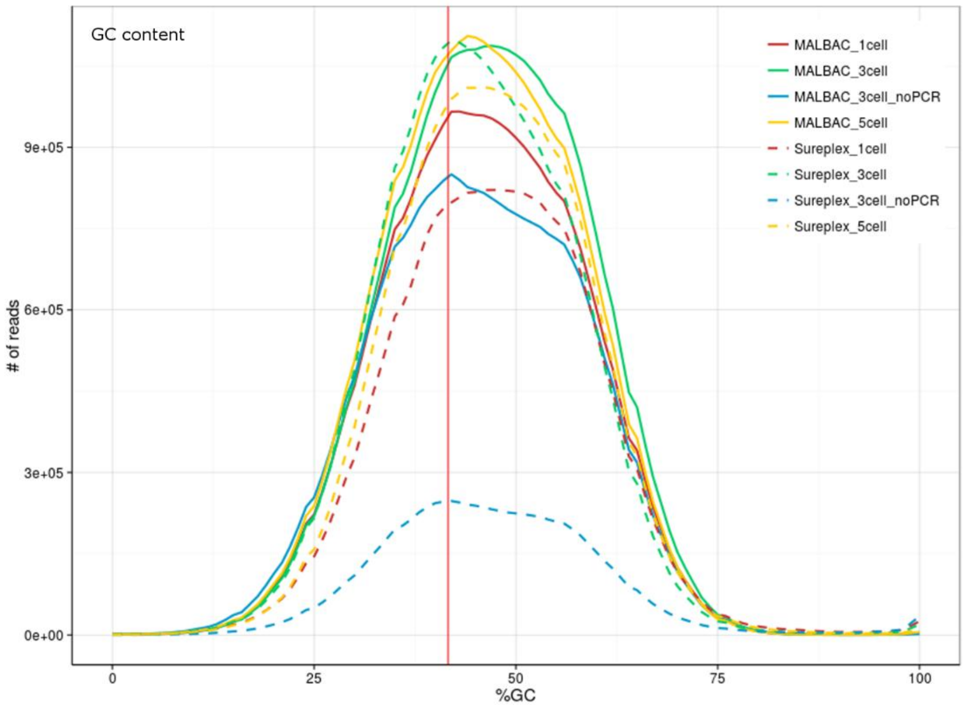

Supplementary fig. S3: Distribution of GC content per read

MALBAC and SurePLex seems to prefer more GC-content rich regions for amplification. Enrichment PCR during library preparation seems to amplify this GC bias effect, i.e. GC-bias is less pronounced in samples that did not undergo enrichment PCR compared to samples that did. However, where usually a clear Gaussian distribution can be expected, the distribution for the samples that were not enriched by PCR is less well-shaped, i.e. no clear maximum/mean can be distinguished. The red vertical line shows the normal GC content of the human reference genome.

Supplementary figure S4

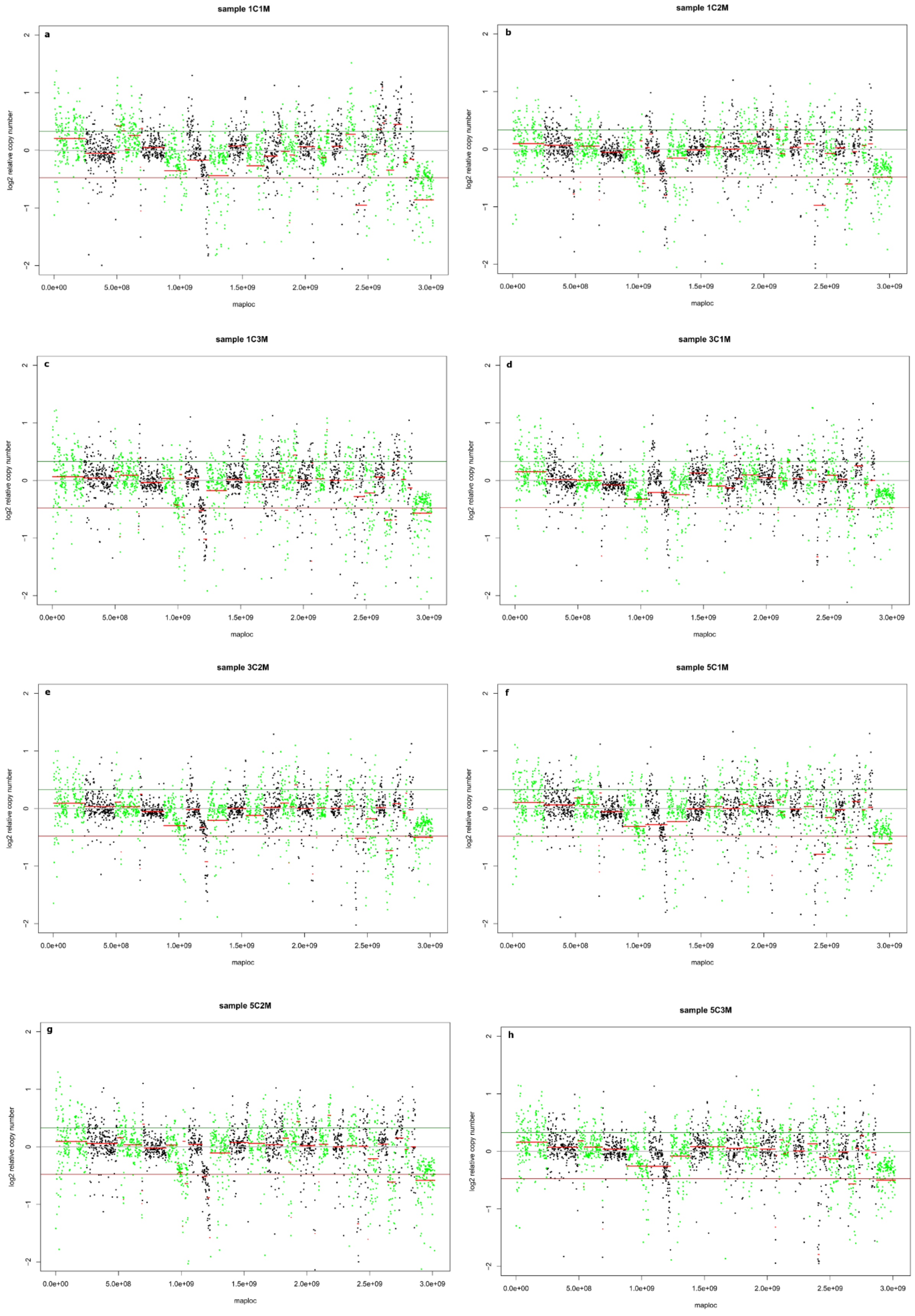

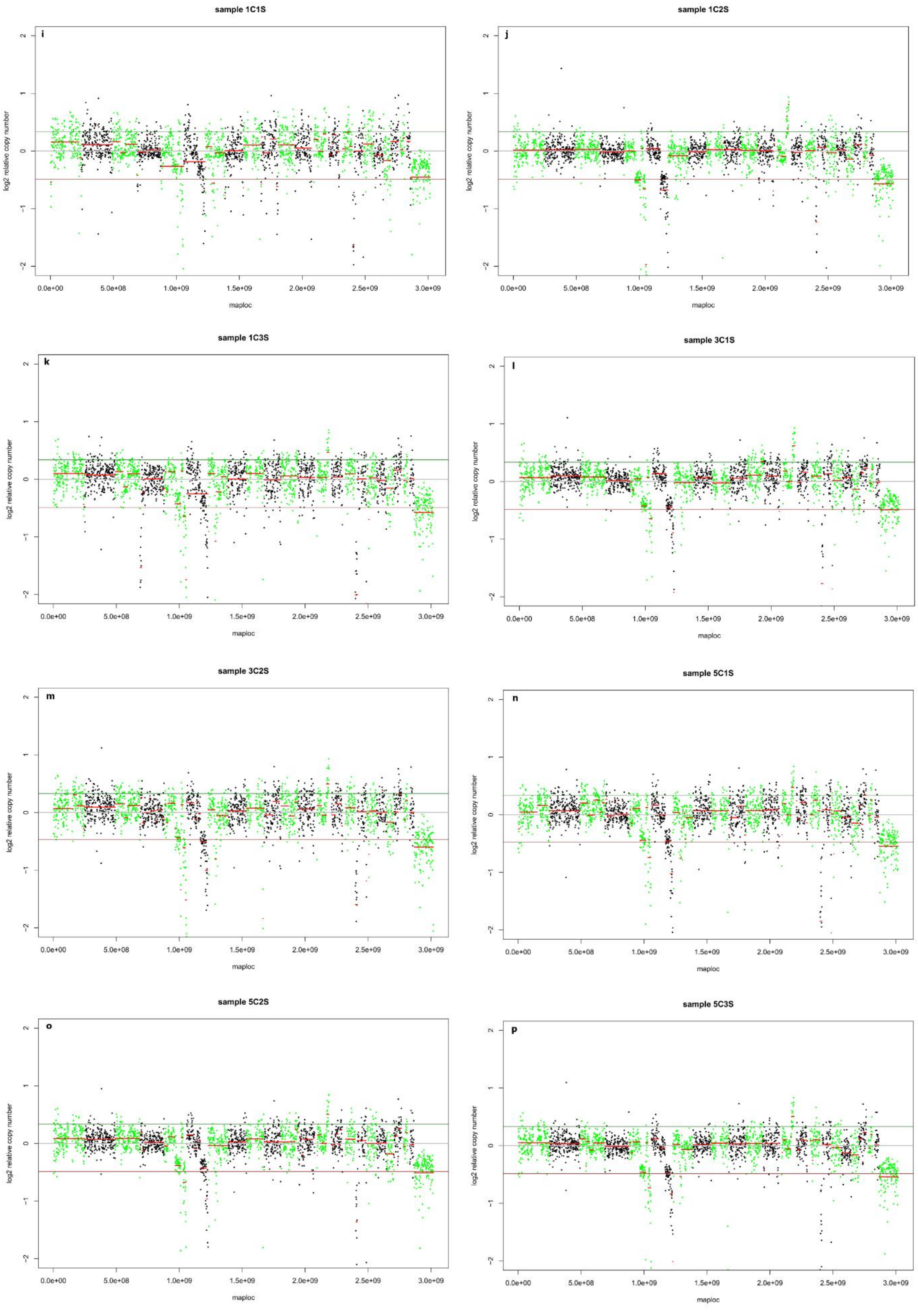

sample 1C1S
i
sample 1C2S
j
sample 1C3S
k
sample 3C1S
l
sample 3C2S
m
sample 5C1S
n
sample 5C2S
o
sample 5C3S
p
log2 relative copy number
maploc
0.0e+00
5.0e+08
1.0e+09
1.5e+09
2.0e+09
2.5e+09
3.0e+09

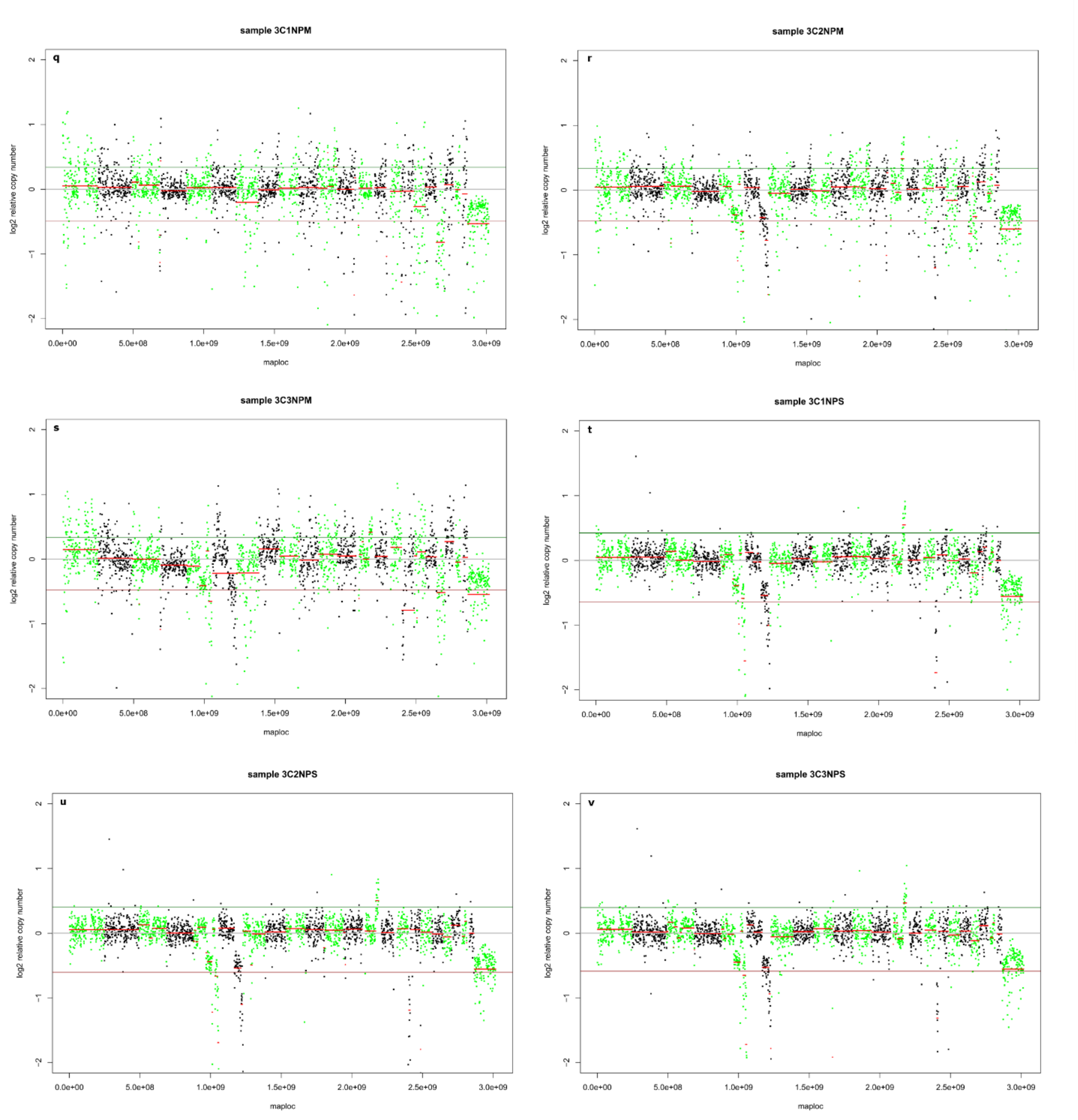

Supplementary fig. S4 : Overview of the CNA profiles of all samples

The CNA profiles of all samples used in this study are shown here. These CNA profiles are similar to the profiles shown in figure 6. The very low copy number of the last part of chromosome 9 will fall out of the range of these graphs, but is present in al SurePlex samples.

Supplementary table S1

List of all CNA detected by ReadDepth

CNA given in grey are the ones that are expected to be detected. The CNA given in de white columns are false positive calls if compared to the reference array.

	detected in																								
**aberration**	**aCGH**	**1cell MALBAC rep1**	**1cell MALBAC rep2**	**1cell MALBAC rep3**	**3cell MALBAC rep1**	**3cell MALBAC rep2**	**3cell MALBAC rep3**	**5cell MALBAC rep1**	**5cell MALBAC rep2**	**5cell MALBAC rep3**	**1cell SurePlex rep1**	**1cell SurePlex rep2**	**1cell SurePlex rep3**	**3cell SurePlex rep1**	**3cell SurePlex rep2**	**3cell SurePlex rep3**	**5cell SurePlex rep1**	**5cell SurePlex rep2**	**5cell SurePlex rep3**	**3cell MALBAC noPCR rep1**	**3cell MALBAC noPCR rep2**	**3cell MALBAC noPCR rep3**	**3cell SurePlex noPCR rep1**	**3cell SurePlex noPCR rep2**	**3cell SurePlex noPCR rep3**
5q-	✓		✓	✓					✓		✓	✓	✓	✓	✓	✓	✓	✓	✓		✓	✓	✓	✓	✓
6q-	✓		✓	✓		✓	✓		✓			✓		✓	✓	✓	✓	✓	✓		✓		✓	✓	✓
9q-	✓							✓	✓	✓	✓	✓	✓	✓	✓	✓	✓	✓	✓		✓			✓	✓
13q+	✓		✓	✓	✓	✓	✓	✓	✓	✓	✓	✓	✓	✓	✓	✓	✓	✓	✓		✓	✓	✓	✓	✓
16p-	✓				✓		✓		✓	✓	✓	✓	✓	✓	✓	✓	✓	✓	✓				✓	✓	✓
Xmono	✓	✓	✓	✓	✓	✓	✓	✓	✓	✓		✓	✓	✓	✓	✓	✓	✓	✓	✓	✓	✓			
1p-											✓														
2q-			✓																						
3p+		✓																							
3p-			✓			✓	✓	✓	✓	✓										✓	✓				
4p+									✓																
4p-													✓												
7m-											✓		✓		✓		✓		✓						
8q+				✓																					
10q-											✓														
10m+					✓																				
11q+				✓		✓	✓	✓	✓	✓				✓						✓					
11m-		✓		✓				✓	✓																
12q+			✓																						
12q-						✓		✓				✓		✓				✓	✓						
13p-		✓			✓																				
15q-																				✓					
16p-							✓																		
16mono			✓			✓		✓														✓			
18m+															✓										
18tris		✓																							
19p-																					✓				
19mono				✓	✓	✓	✓	✓	✓	✓										✓		✓			
20q+			✓	✓													✓								
20m-				✓			✓																		
20tris		✓																							
